# Predicting Future Conversion from Amyloid‐β Negativity to Positivity: Time‐To‐Event Prediction with Machine Learning

**DOI:** 10.1002/alz.093174

**Published:** 2025-01-09

**Authors:** Amirali Vahid, Jafar Zamani, Hadi Hosseini

**Affiliations:** ^1^ Stanford University, Stanford, CA USA

## Abstract

**Background:**

Alzheimer's disease (AD) is characterized by the gradual accumulation of amyloid‐β (Aβ) plaques and tau tangle pathologies in the brain. Extant studies highlight that Aβ deposition contributes significantly to tau accumulation, leading to neurodegeneration and cognitive decline. Individuals with positive Aβ are more susceptible to AD progression but the precise spatial and temporal pattern of Aβ leading to convergence from negative to positive status is unclear. This study aims to predict the likelihood and the number of years it would take for an individual to transition from baseline negative Aβ to positive (i.e., transition time) using Aβ PET measures with machine learning methods for time‐to‐event and to further identify the most predictive brain regions.

**Method:**

PET measures for Aβ SUVR of 446 subjects (216 females, mean age 71 ± 7), initially presenting negative Aβ from the Alzheimer’s Disease Neuroimaging Initiative (ADNI), were utilized. The subjects had a longitudinal follow‐up averaging 4.76 ± 3.5 years, ranging from 1 to 11 years, with 86 transitioning to positive Aβ. For time‐to‐event analysis, three machine learning models— CoxPH, Random Survival Forest (RSF) and Survival XGBoost—were employed to predict the transition time. Their performance was assessed using 5‐fold cross‐validation and evaluated by the concordance index (C‐index). The permutation method was used to determine feature importance as an explainable AI approach.

**Result:**

The C‐index, were 0.71, 0.834, and 0.829 for CoxPH, RSF, and Survival XGBoost, respectively. In the RSF that resulted in the highest accuracy the permutation method highlighted the significant influence of Aβ load in the rostral/medial frontal regions, inferior temporal region and precuneus. Post‐hoc correlation analyses confirmed these results and showed significant negative correlations between Aβ load in these regions and the transition time. Furthermore, based on the Kaplan‐Meier estimate, the median duration for transition time was 3.94 years.

**Conclusion:**

Elevated Aβ levels in the medial and rostral frontal regions strongly predict the time of transition from negative Aβ to preclinical AD (i.e., positive Aβ). These findings highlight the potential of these regions as biomarkers for transition to preclinical AD, emphasizing their prospective application in AD therapy and drug discovery.

